# Development of Oil Industry in Poland in the Context of the European Union

**DOI:** 10.3390/foods13213406

**Published:** 2024-10-25

**Authors:** Aneta Bełdycka-Bórawska, Piotr Bórawski, Lisa Holden, Tomasz Rokicki

**Affiliations:** 1 Department of Agrotechnology and Agribusiness, Faculty of Agriculture and Forestry, University of Warmia and Mazury in Olsztyn, 10-719 Olsztyn, Poland; pboraw@uwm.edu.pl; 2 Department of Animal Science, Faculty of Agricultural Sciences, Pennsylvania State University, University Park, PA 16802, USA; lah7@psu.edu; 3 Management Institute, Warsaw University of Life Sciences, 02-787 Warsaw, Poland; tomasz_rokicki@sggw.edu.pl

**Keywords:** fat, industry, EU, economic situation

## Abstract

Oil industry is developing well in Poland. This study aimed to examine the development of the Polish oil industry after joining the European Union. The following questions were asked: What were the changes in the consumption of vegetable oils in Poland after EU enlargement in 2004? How did the production of vegetable oil change in Poland after EU enlargement in 2004? And has the liquidity of the Polish oil industry changed after EU enlargement in 2004? First, the changes in the oil industry were evaluated. This research proved the development of the fat industry. We used the regression model to analyze the impact of chosen factors on the economic situation of the oil industry in Poland. Rapeseed is the main source of oil for the edible oil and biodiesel industry. High prices of rapeseed increased the sown area of rapeseed. The production of crude rapeseed oil changed from 520.1 thousand tons in 2005 to 1440 thousand tons in 2023. Biofuels made from rapeseed oils are called first generation. Poland’s production of refined rapeseed oil changed from 386.5 thousand tons in 2005 to 541 thousand tons in 2023. These numbers confirm the development of edible oil and biofuel production. The gross profit increased from PLN 133 mln in 2005 to PLN 443.8 mln in 2023. The net profit of the fat industry increased from PLN 110 mln in 2005 to PLN 381.6 mln in 2023. The oil industry is characterized by high investment, which reached the level of PLN 232.9 mln in 2023. The investment rate in 2023 was PLN 1.96 %. The changes observed in the rapeseed market after Poland joined the EU affected the performance of vegetable oil and biofuel producers. The regression analysis proved the hypothesis that the monthly consumption of rapeseed oil is the main factor influencing the oil industry situation in Poland. The oil industry is important to the development of the whole economy. Particular attention should be paid to current liquidity, which is why a constant supply of money from sold oil and biodiesel is needed to cover costs.

## 1. Introduction

Currently, consumers are demanding and requiring a healthier diet. An important part of a healthier diet is nutrition, including oils and fats [1]. Foods containing increased amounts of polyunsaturated fats have been commercialized [2].

Rapeseed, palm, soybean, sunflower, and linseed oils have many applications. They are used in oil, petrochemical, food, and other industries. Moreover, these plants deliver lignocellulosic biomass used in different industries [3]. The most used vegetable oils in 2017–2018 were rapeseed (39%), followed by palm (25%). The least used were sunflower oils (18%) and soybean oils (9%) [4].

The European Union (EU) is the main producer of rapeseed oil and is importing high levels of soybean oil for energy purposes. The increasing demand for biofuels and ambitious EU regulations caused high prices of rapeseed and soya in the market [5].

Rapeseed is called canola in the United States of America (USA) and in Canada. It belongs to the Brassicaceae family, and it has high yields worldwide [6,7,8,9].

Rapeseed oil (canola) is known for its rich content of unsaturated fatty acids, including linoleic (omega-6) and α-linoleic acid (omega-3), and low levels of saturated fatty acids [10]. Because of the EU’s energy policy focusing on the use of renewable energy sources including biodiesel, rapeseed (canola) production increased to 72.3 million tons in 2020 compared to 29.7 million tons in 1994 [11].

Rapeseed (canola) and its prices are the main effects of the volatility of all vegetable oils. The shock in crude oil markets caused the uncertainty in vegetable oil prices, thus affecting the economic situation of the enterprises processing rapeseed [12].

Palm oil is of great importance in the food industry [13]. Due to the high levels of palmitic acid, palm oil has been used for margarines and shortenings [14]. Palm oil is grown worldwide, including 40 countries, especially in South Asia [15,16,17].

Soybean oils and coconut oils are produced mainly in the USA, and they differ from rapeseed oil. The unsaturated fatty acids are abundant in soybean oils, while saturated fatty acids are plentiful in coconut oil. There is a significant difference in the fatty acid composition between soybean oil and coconut oil. In terms of morphology, color, and fibrous structure, the coconut oil group performed better than soybean oil [18].

Sunflower oil is also produced in the EU and other areas around the world. The components of sunflower oil include phospholipids, free fatty acids, peroxides, aldehydes, trace metals, polymers, waxes, mono- and diacylglycerols, and moisture, which are not necessary and should be eliminated in the refining process [19,20]. The sensory properties, the hydrolytic and oxidative stability of refined oil, as well as oil losses during refining are all negatively impacted by these components [21,22,23].

Rapeseed, palm, soybean, and sunflower oils are the main products for utilization and processing in the fat industry in the European Union (EU). The fat industry in Poland is mainly processing rapeseed for edible oils and biodiesel. The fat industry plays an important role in the Polish agri-food sector. There were 17 fat-producing and processing companies in Poland in recent years. Most of them supply rapeseed oil for food and biodiesel production. Between the years 2001–2003 and 2014–2016, rapeseed processing and the production of crude rapeseed oil increased from 320,000 to 1.22 million tons, whereas margarine production increased from 370,000 to 380,000 tons, respectively. In 2021, the production of crude rapeseed oil reached the level of 1331.8 million tons [24]. Agriculture is an important producer of not only food products but also the biomass used for energy purposes and the production of energy from renewable energy sources. It leads to the development of a circular economy [25]. The economy promotes the utilization of sustainable and renewable bioresources coming from the agriculture and industrial sectors [26].

The literature concerning rapeseed production, which is the main product of biodiesel and edible oils production, is well described.

However, little attention is paid in the literature to the development and economic situation of the fat industry in Poland, which is processing rapeseed. The intention of the authors of this paper was to fill in the existing research gap.

This study aimed to examine the development of the Polish oil industry after joining the European Union. The following questions were asked:

1. What were the changes in the consumption of vegetable oil in Poland after EU enlargement in 2004?
2. How did the production of vegetable oil change in Poland after EU enlargement in 2004?
3. Has the liquidity of the Polish oil industry changed after EU enlargement in 2004?

The following hypothesis was formulated:

**Hypothesis 1 (H1).**  *Monthly consumption of rapeseed oil is the main factor affecting the economic situation of the oil industry in Poland.*

This text is divided into five sections. The first part is the Introduction. Materials and Methods are described in other sections. The third section describes the Results, which are discussed in the fourth section. The Conclusion is collected in the last part of this article.

### Factors Shaping Economic Situation of Oil Industry in Poland in the Context of the EU

The performance of the Polish fat industry is unstable. After a brief period of improvement in 2013–2014, market performance decreased in 2015–2017. Domestic demand for edible rapeseed oil decreased from 2004 to 2017. However, rapeseed production and processing improved in Poland from 2005 because the EU implemented new policies regarding biodiesel and renewable energy.

The development of new energy sources, including biofuels, is known in the literature. However, little attention has been paid to the power and development of the oil and biofuel industry.

The economic performance of the fat sector changed in 2005–2023. The analyzed companies posted profits in 2005–2010 and 2013–2017 but reported losses in 2011–2012. In turn, biofuel producers reported losses in 2008 and 2014 but achieved good economic results in the remaining years of the studied period.

Investments in the oil and biogas sector increased significantly after Poland joined the EU. As a new member state of the EU, Poland had to quickly adapt its agricultural and food industry to EU standards, which required significant investment [27].

The production of rapeseed is mainly located in China, India, Canada, and the EU. Rapeseed is processed as different products such as oil, which is the most important product for consumers in the EU. The biggest consumption of another product, which is rapeseed meal, is also in the EU [28].

In the EU, 77% of biofuel is produced from first generation, which includes mostly oil, rapeseed. Production in the EU is diversified, and Germany occupies first place as the biggest producer, followed by France, Poland, and the Czech Republic [29].

The main reason for increasing interest in rapeseed production is the increasing price of petroleum. The increasing price of petroleum is the effect of a higher demand for petroleum and energy in countries. Fossil fuels are responsible for carbon emissions, and there is an increased need to look for more environmentally friendly fuels, like biodiesel made from rapeseed [30,31,32].

The EU has undertaken an ambitious goal to increase the share of biofuels for 2010 by 5.75%. However, this goal is very difficult to achieve because of the strong competition for rapeseed in the market [33]. That competition is why the European Commission allowed the use of different products to achieve more biofuels. Norway uses residual animal fat and fish for biodiesel production [34]. Even though Norway is not a member of the EU, its experience in this field may be useful for other countries.

Bełdycka-Bórawska [35] analyzed the changes in the biofuel industry in Poland after joining the EU. The author found that Poland has increased rapeseed usage for biodiesel production compared to edible oils. The share of the vegetable and animal oil fat industry is 4.63% in the food industry. The demand for margarine and edible fat is small because consumers use more liquid oils, and that demand is why there is a small number of companies in this area [35].

In recent years, the development of the food industry caused an increase in competitiveness. This competitiveness happened because of modernization and investments in the food industry. The investments were particularly noticeable in fixed assets [36]. Oil and fat enterprises are important parts of the food industry in Poland. The oil industry in Poland is characterized by a high degree of production concentration. Thirteen companies operate businesses, employing from 8 to over 50 people. The four largest companies (Kruszwica, Brzeg, Szopienice and Warsaw) produce over 80% of oils and margarines. The main factor in the development of this industry was the increase in demand from Polish consumers for vegetable fats, related to the change in diets. Therefore, the domestic market has become the main recipient of this industry (95% of sales).

The state of the Polish oil industry depends on the access of rapeseed oil. The area of rapeseed production in Poland increased from 946.1 thousand hectares in 2010 to 1102.6 thousand hectares in 2023. It created the surplus of rapeseed, and the production was 3739.5 thousand tons in 2023 compared to 2228.7 thousand tons in 2010 [24]. Fertilization is important in shaping rapeseed yields. Proper agrotechnics, especially fertilization and the application of plant protection products, contribute to their optimal use, while reducing the risk to natural environment. Too much fertilization has no significant impact on yield and causes the growth of unused ingredients by plants [37,38].

## 2. Materials and Methods

### 2.1. Data Sources

The oil industry performed better, and jobs in that sector increased after Poland joined the EU [6]. Employment decreased from 3300 to 3000 between 2008 and 2011, but it increased rapidly to 5200 in 2013 (by 57.6% between 2008 and 2013). Employment was highest in large and medium-sized companies. In these companies, employment increased from 3200 to 4700 between 2008 and 2013.

Current assets in the oil industry were highest in 2014 (PLN 2,182,000 million) and 2009 (PLN 2,122,000 million), and lowest in 2011 (PLN 768,600 million), 2017 (PLN 1,121,000 million), and 2016 (PLN 1,150,000 million). Data for this study were obtained from the financial statements of selected oil and biofuel producers [24].

### 2.2. Methods

The authors of this paper analyzed the factors shaping the Polish economy in the oil industry. To analyze the factors influencing the gross profit of the examined oil producers, the best method is regression analysis. The following equation was used to estimate the regression model:

Yi = *α* + *β*_1_∙x_1_ + *β*_2_∙x_2_ + … + *β*_i_∙x_i_ + *ε*_i_ for i = 1, 2, 3…n

where:

Y—endogenous variable,
x—exogenous variable,
*α*—permanent,
*β*_1_, *β*_2_…—regression,
*ε*_i_—irregular variable.

The impact of particular variables on the regression equation is used by partial and semi-partial correlations [39,40]. The distinct contribution of an independent variable to the overall variance in the dependent variable is measured by a partial correlation. By accounting for the influence of additional independent variables on the dependent variable, a squared partial correlation calculates the residual variation in the dependent variable that is explained by the independent variable. Conversely, a squared semi-partial correlation calculates the entire variance explained by a certain independent variable, but a semi-partial correlation does not take into consideration the impact of other factors on the dependent variable. The F-test was used to measure the regression model’s overall significance.

The authors of this paper calculated the significance of regression coefficients (Student’s *t*-test), statistical significance (*p*-value at *p* ≤ 0.05, standard error of the mean, coefficients of determination (R^2^)), and correlation coefficients. The model was a forward stepwise regression [41,42]. The sets of variables were limited to a few that had a statistically significant effect on the gross profit of oil producers.

Gross profit Y_1_ was chosen in order to determine the market variables influencing fat manufacturers’ performance, and Y1 was employed as the dependent variable. This parameter was selected for analysis due to the availability of the relevant data. The variable Y_1_—the gross profit of the examined oil producers was directly influenced by variables X_1_–X_8_. The following factors affected gross profits in the oil and fat industry:

X_1_—domestic rapeseed production,
X_2_—rapeseed processing,
X_3_—margarine production,
X_4_—price of rapeseed oil,
X_5_—current liquidity,
X_6_—price of exported rapeseed,
X_7_—monthly consumption of rapeseed oil,
X_8_—monthly export of rapeseed oil.

## 3. Results

### 3.1. Production of Vegetable Oil

The production of vegetable oil (in ′000 tons) in companies employing 10 or more workers is presented in Table 1. Rapeseed oil is important because it is the second leading culinary oil produced globally. Moreover, it has healthy ingredients such as unsaturated fatty acids and bioactive components. In addition, the consciousness of consumers is increasing, which creates the demand for rapeseed [43].

The production of vegetable oil increased at the highest rate between 2005 and 2023. In 2023, the production of crude rapeseed oil (for food processing and industry) reached 1440.0 million tons, and the production of refined oils reached 547.5 million tons [24]. Between 2005 and 2023, the production of crude rapeseed oil increased by 176.9%, the production of rapeseed oil increased by 101.9%, the production of refined oil increased by 40.1%, and the production of edible rapeseed oil increased by 110.1%. Edible soybean oil was not produced in the time period 2014–2016. In turn, the production of edible sunflower oil decreased by 76.7% between 2005 and 2023 (Table 1).

Higher rapeseed production increased rapeseed processing to meet the growing demand for biofuels in Poland and the EU [24]. The volume of processed rapeseed increased from more than 300,000 tons before Poland’s accession to the EU to more than 1 million tons on average in 2013–2023. The performance of agricultural holdings producing oilseed crops was influenced by the demand for biocomponents for biodiesel production. According to the Renewable Energy Directive (RED), 10% of the liquid fuels for transport should be derived from renewable sources by 2020.

### 3.2. Financial Performance of Polish Fat Producers

Revenues in the oil industry fluctuated between 2005 and 2023. Revenues were highest in 2022 (PLN 13,289.9 million), 2023 (PLN 10,447.6 million), and 2021 (PLN 8560 million), and they were significantly lower in 2005 (PLN 2571 million) and 2006 (PLN 3237 million). Net revenues accounted for 95.2% in 2011 and decreased to 80.4% in 2021.

The financial performance of vegetable oil producers was evaluated based on their gross and net profits. Both gross and net profits were negative in 2011–2012 (Table 2), and the performance of the vegetable oil sector decreased due to a decline in output and rapeseed processing, as well as an increase in raw material costs relative to the price of the final product [44]. This downturn could also be attributed to the global financial crisis and economic slowdown in countries that import Polish goods [45].

Vegetable oil producers improved their performance and reported positive gross and net profits in 2005–2010 and 2013–2017. Gross and net profits peaked in 2014 (PLN 519.7 million and 451.8 million, respectively) and 2021 (PLN 451.9 million and 378 million, respectively).

**Figure 1:**
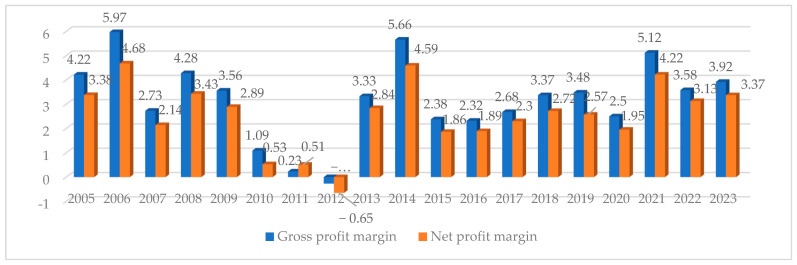
Gross and net profit margin of fat industry in Poland (%). Source: own elaboration based on [38].

The economic situation of the oil industry was also assessed based on profit margins, namely, the ability to generate positive financial results. The gross profit margin fluctuated in the analyzed period (Figure 1), and it was determined at 5.97% in 2006. However, negative values were reported in 2012. In this period, vegetable fat producers reported losses due to a steep increase in operating costs, and revenues did not exceed expenses.

In turn, the net profit margin peaked in 2006 and 2014 and was lowest in 2011 and 2012. In 2011 and 2012, the net profit margin in the fat industry was negative at −0.65.

**Figure 2:**
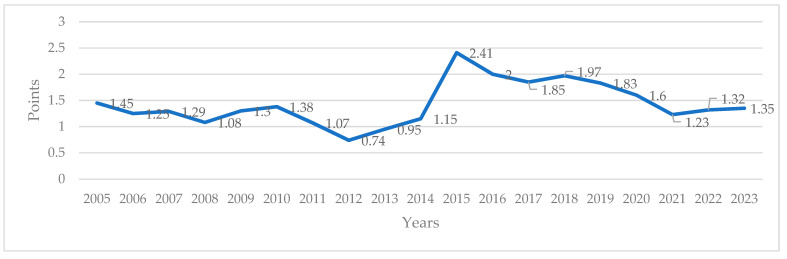
The current liquidity ratio in the fat industry. Source: own elaboration based on [24].

Current liquidity, specifically, a business’s capacity to pay its short-term debt, is yet another factor that influences financial performance. Liquidity is affected by numerous factors, including the source of funds for financing assets and debts [46,47]. According to the literature, the current liquidity ratio should range from 1.5 to 2 [48]. Values below 1.3 indicate that a company does not have enough liquid assets to cover its short-term liabilities. In the Polish oil industry, the current liquidity ratio, namely, the ratio of short-term liabilities to current assets, fluctuated between 2005 and 2023. In 2005–2014, the analyzed parameter was below the recommended value. The situation changed in 2015 when the current liquidity ratio reached 2.41. In the following years, the examined parameter did not decrease below two (Figure 2). The liquidity ratio was lowest in 2012 (0.74) and 2013 (0.95), when oil producers experienced problems with meeting their current liabilities. Low liquidity can increase financial costs and discourage investors from trading a company’s assets [49].

The current liquidity was highest in 2015 (2.41), 2016 (2.0), and 2018 (1.97), when surplus cash inflows were reported (Figure 2). In these years, oil producers maintained a healthy ratio of cash inflows to outflows. The current liquidity decreased to 1.23 in 2021 [50].

Between 2005 and 2013, the oil and fat industry’s investments grew in value from PLN 71.5 million to PLN 243 million (by 243%). However, investments decreased to PLN 68 million in 2017 (by 72.10%). The investment value in the oil sector was highest in 2012 (PLN 292 million) and 2013 (PLN 243 million), and lowest in 2017 (PLN 68 million), 2005 (PLN 71.5 million), 2006 (PLN 76 million), and 2015 (PLN 77 million) (Table 3). According to Łącka [45], at the beginning of Poland’s accession to the EU, investments in the food processing industry (including the oil and fat sector) were driven by high domestic demand and food exports.

The investment rate in the oil industry was highest in 2011 (1.24%), 2012 (1.44%), and 2023 (1.96), and lowest in 2015 (0.61%) and 2010 (0.75%) (Table 3). Investment fluctuations in the food processing industry (including the oil and fat sector) could be attributed to an economic slowdown, high prices in the domestic and global market, which decreased demand for food, and the Russian embargo on EU food imports introduced in 2014 [45].

In summary, investments in the Polish oil sector improved labor productivity, enabling companies to purchase modern equipment and generate a production surplus. According to Łącka [45], in the coming years, the value of investments in the Polish oil and fat industry will be determined by exports, in particular by non-EU markets, product innovation, mergers and acquisitions, and investments in human capital.

Many companies rely on external sources of funding, such as short-term and long-term loans, to finance their development. Businesses can opt for various sources of funding, but the proportions of equity capital and borrowed capital must be maintained at an optimal level. High debt carries financial risks, and it can compromise a company’s performance [51,52]. The cost of debt and the availability of external funding are the main criteria that influence a company’s financial decisions [53]. Short-term loans are used to finance current operations, whereas long-term loans support investment and business growth. In companies under study, short-term liabilities were highest in 2022 (PLN 2220 million), 2023 (PLN 1813 million), and 2013 (PLN 755 million), and lowest in 2016 (PLN 92 million) and 2017 (PLN 112.1 million) (Table 4).

Short-term and long-term debt in the fat industry are presented in Table 4. The ratio of short-term debt to current assets improved in 2005–2023. The analyzed parameter decreased from 0.81 in 2012 to 0.06 in 2017 due to an increase in current assets. The ratio of short-term liabilities (excluding loans) to current assets decreased from 0.65 in 2011 to 0.28 in 2015. In turn, the ratio of long-term debt to depreciation grew higher, and it reached 2.2 in 2010. These observations indicate that long-term liabilities enabled vegetable oil and fat producers to invest in fixed assets.

### 3.3. Regression Analysis of Factors Shaping the Economic Performance of the Polish Fat Industry

A multiple regression analysis was used to ascertain how much the state of the rapeseed market influenced the performance of fat producers.

The results are presented in Table 5, Table 6 and Table 7. The dependent variable was the gross profit of vegetable oil producers—Y_1_. Dependent variables were described in the Section 2.2.

The analysis revealed that the gross profit of vegetable oil producers was influenced by the following variables (Table 5): X_1_—domestic rapeseed production, X_3_—margarine production, X_5_—current liquidity, X_6_—price of exported rapeseed, X_7_—monthly consumption of rapeseed oil, and X_8_—monthly export of rapeseed oil. Two variables, X_2_—rapeseed processing and X_4_—price of rapeseed oil, were not important in the model. The final regression can be described by the following equation:

Y = 14.443 + 0.058X_1_ − 1.200X_3_ − 3.200X_5_ − 0.060X_6_ + 4.110X_7_ + 0.031X_8_

The observed data were well fit by the regression model (R^2^ = 0.998; R^2^ = 0.99733886, df = 6.13, F = 939.8120).

The regression analysis findings show that, at a significance level of *α* = 0.05, there is no reason to reject the null hypothesis. This finding suggests that the slope of the regression line is statistically significant and that some in-dependent variables have a significant correlation with gross profits in the vegetable oil and fat industry (Table 6).

To ascertain how each independent variable affected the dependent variable, semi-partial correlations were computed (by eliminating the effects of the remaining variables). A semi-partial correlation provides information about the extent to which an independent variable influences the dependent variable, but it only explains the proportion of variance that is not explained by other analyzed variables (predictors). Therefore, a semi-partial correlation provides information about the magnitude of the unexplained (residual) variance of the dependent variable in the model [53,54]. The highest values of the coefficient of semi-partial correlation were noted for variables X_7_—the monthly consumption of rapeseed oil and X_5_—current liquidity, which indicate that these variables exerted the strongest effects on the dependent variable (Table 7).

## 4. Discussion

There are several uses for vegetable oils. They are employed in the food processing sector to create canned goods, edible oils, butter, and margarine. Because of their high protein content, rapeseed meal and oil cake are utilized to make animal feed concentrates. Following some nations’ bans on the use of meat and bone meal in the manufacturing of animal feed, there was a surge in demand for rapeseed meal and oil cake [55]. Additionally, vegetable oils have wide use in the chemical sector, where they are used to make oil-based paint, soap, detergents, varnish, and soap. Biodiesel esters are produced from rapeseed in the petrochemical sector [56].

The food industry is developing well in Poland. However, the main factor in the growth of the food industry was exports, supported by a growing domestic demand [57]. The Polish food industry generates about EUR 7.5 billion of the gross added value, which constitutes more than 4% of the GDP [58]. Part of the food industry, the oil industry plays a very important role, and the functioning of oil and fat has a significant role in determining the financial health of several businesses, both in and outside of agriculture [59].

It was proven that the production of crude oil increased in Poland after accession to the European Union. The increase rate of crude rapeseed oil in 2005–2023 increased by 176.9% and rapeseed oil by 101.9%. Such an increase could be achieved by the increase in the sown area of rapeseed in this period and the increase in imports.

Domestic consumption of vegetable oil is the most important factor determining the changes in the oil and fat industry in Poland. The consumption of vegetable oil increased by 75% between 1961 and 2015, which shows global changes in the market and the role of consumers [60].

The financial performance of vegetable oil was generally positive in 2005–2023. However, in 2011–2012, gross and net profits were negative. This slowdown trend could be the effect of financial crises, which had a negative impact on the economic situation of enterprises in the world.

Among the factors shaping the economic situation of the oil industry is the trade balance of products. Poland had negative trade balances for vegetable oil in 2017–2023, reaching the level of EUR −840 mln. Only the trade balance of rapeseed oil was positive in 2023, reaching the level of EUR 40.9 mln. These results demonstrate that the demand for vegetable oil is increasing [61].

The Polish oil industry is characterized by a small number of companies. The oligopolistic tendencies in the Polish oil and fat industry create the risk for concentration in the market. Such a situation leads to the fact that the majority of revenues are generated by the main ten companies [62].

The overall growth of the Polish oil industry is determined by regional production. The production of rapeseed, which is the main product, is located in regions with big farms; however, its production is expanding into new areas. The possibility of using more land for purposes under the EU support program, which initiated expansion, and the expansion have continued after the program expired [63].

Another factor determining the economic performance of the Polish oil industry is the production of biodiesel, as rapeseed is the main source of products for biodiesel production in Poland and other European Union (EU) countries (Germany, Spain, France, the Netherlands, and Poland). Such a situation increases the demand for rapeseed and prices of imports [64].

The development of the oil sector will depend on prices of raw material and retail prices of edible oil and biodiesel. This relation is called price transmission, and the access of cheap rapeseed impacts the retail prices of oil and biodiesel [65].

Biodiesel is becoming more important in achieving sustainable development goals. Moreover, biodiesel production in the EU is strongly correlated with first generation biofuels prepared mainly from rapeseed [66].

The development of renewable energy sources will increase competitiveness [66]. The production of biodiesel from rapeseed improves the security of the energy sector in the European Union (EU). The efficient use of resources helps to achieve Sustainable Development Goals (SDGs) [67,68,69].

The growth of the oil industry in Poland and other EU member states is dependent upon Information and Communication Technology (ICT), which helps to promote governance and citizen participation in the public decision-making process. Such a process is taking part in the European Union (EU). Important links are between the democratic quality of countries and the levels of digital transformation, which increase productivity in all sectors, including the oil sector [70].

## 5. Conclusions

The Polish oil industry is developing well after accession to the EU. It was the result of the utilization of fat oils in the nutrition and petrochemical industries. The Polish oil industry is well concentrated and includes 13 companies. However, the vast majority of Polish oils and margarine are produced by four main companies (Kruszwica, Brzeg, Szopienice and Warsaw).

The production of vegetable oils in Poland increased in 2005–2023. The highest levels were achieved in 2023, when the production of crude rapeseed oil (for food processing and industry) reached 1440.0 million tons, and the production of refined oils reached 547.5 million tons. The situation of the Polish fat industry was characterized by the fact of a constant increase from 2005 to 2023, when the production of crude rapeseed oil increased by 176.9%, the production of rapeseed oil increased by 101.9%, the production of refined oils increased by 40.1%, the production of edible rapeseed oil increased by 110.1%, and the production of edible sunflower oil decreased by 76.7%.

The changes observed in the rapeseed market affected the performance of vegetable oil and biofuel producers. Gross and net profit margins in the vegetable oil sector were negative only in 2011 (−0.23% and −0.51%, respectively) and were positive in the remaining years of the analysis. In turn, biofuel producers embarked on large investment projects to upgrade their plants and equipment, which compromised their financial performance in 2008 and 2014.

One of the most important factors shaping the development of the oil industry is investment. The data proved that the Polish oil sector increased the investment; however, the levels were different in particular years. The biggest investments were in 2012 and reached the level of PLN 292 million and the lowest in 2017 (PLN 68 million). Then, the investment started to grow and reached the level of PLN 232.9 million in 2023. The changes in investment in the oil sector were the effect of the general conditions of the economy of the European Union (EU). The investment rate also changed in 2005–2023. It was the smallest in 2010 (0.75%), then started to grow, and reached a level of 1.96% in 2023.

Short debts to current assets also changed in 2005–2023, showing improvements in the economic situation of the sector. The short-term loans reached the highest level in 2022 (PLN 2220 million) and 2023 (PLN 1816 million), which proves that the oil sector needed external money for the current development. Long-term loans were the highest in 2012 (PLN 481.4 million) and 2019 (PLN 312.3 million). These results prove that long-term loans increased in accordance with programs of the EU in the periods 2007–2014 and 2015–2020. The oil industry needed extended money to finance investments.

An important factor describing financial performance is the liquidity ratio. The liquidity ratio was lowest in 2012 (0.74) and 2013 (0.95), causing an increase in financial costs. The current liquidity was highest in 2015 (2.41), 2016 (2.0), and 2018 (1.97), when surplus cash inflows were reported.

Based on our research, we confirmed the Hypothesis 1 (H1) that the ***monthly consumption of rapeseed oil is the main factor affecting the economic situation of the oil industry in Poland.*** These results confirm that the significant role of demand is shaping the economic situation of goods producers.

The research results can have limitations. The first limitation can be the access of reliable data. The data of the model were obtained from the rapeseed market [24]; however, monthly data would be more suitable for this research. These data are not available within public statistics.

The authors of this paper analyzed the factor affecting the performance of the oil industry in Poland. However, more data on a European Union level would increase the analysis. These data are difficult to achieve on the EU level.

## 6. Policy Implications

Our research can be useful for policy makers and other information consumers. The oil industry in Poland is important to the development of the whole economy. Particular attention should be paid to current liquidity, which is why a constant supply of money from sold oil and biodiesel is needed to cover the increasing costs of production.

The high supply of the raw material in Poland made the oil industry one of the leading specializations of Polish agri-food processing. Due to a good demand situation of the rapeseed oil market, local producers have increased their activity.

The development of the oil industry is also important for achieving sustainable development goals (SDGs) of Poland and the European Union (EU). More and more rapeseed oil is used for biofuels. The production of rapeseed oil in 2023 was 1,440,000 tons. The production of bio-diesel esters was 971 thousand tons. This amount means that more rapeseed oil is allocated to the production of esters, i.e., sustainable development goals. The foreign trade balance of biodiesel is negative and in 2022 amounted to −125.1 thousand tons, which indicates biodiesel as the future direction of rapeseed oil processing. The EU has established the RED (Renewable Energy Directive), and the production of biodiesel helps to achieve this goal. Poland has great potential for processing rapeseed for biodiesel and edible oil, and it should be the future direction of the development of oil industry. The European Union (EU) policy concerning biofuels will be the important factor stimulating the production of rapeseed in Poland and other countries. This policy aims to increase energy efficiency and security and to reduce the negative impact of the economy and agriculture on the environment.

## Figures and Tables

**Table 1:** Production of vegetable oil and fats (in ′000 tons).

Year	Crude Rapeseed Oil	Refined Vegetable Oil	Refined Vegetable Oil by Type					Margarine
			Rapeseed Oil	Rapeseed Oil by Type		Edible Soybean Oil	Edible Sunflower Oil	
				Edible	Industrial			
2005	520.1	427.4	386.5	286.5		15.1	25.8	347.8
2006	600.4	452.1	378.1	278.1		21.4	52.8	345.4
2007	633.1	502.6	444.4	444.4	-	9.2	49.0	345.1
2008	713.0	640.8	605.9	532.2	73.7	15.5	19.4	341.2
2009	882.6	665.3	633.5	534.0	99.5	9.5	22.3	363.0
2010	817.5	631.4	617.6	474.9	142.7	0.3	13.5	391.3
2011	695.6	610.6	591.4	490.3	101.1	3.1	16.1	401.9
2012	831.8	664.0	636.1	442.2	193.9	0.1	27.8	426.1
2013	1002.3	751.2	735.3	503.2	232.1	0.1	15.8	432.4
2014	1258.1	736.5	720.1	463.5	256.6	0.0	16.3	421.4
2015	1276.6	729.0	714.3	508.3	206.0	0.0	14.6	348.3
2016	1119.5	837.4	819.4	489.0	330.4	0.0	18.0	359.1
2017	1148.7	835.5	813.2	465.3	347.9	0.1	22.2	335.1
2018	1239.3	785.5	756.6	536.5	220.1	0.1	28.8	320.4
2019	1249.7	748.0	715.3	503.3	212.0	0.2	32.5	318.6
2020	1267.4	709.1	674.8	504.2	170.6	0.1	34.3	327.8
2021	1331.8	794.4	780.6	532.0	248.6	0.1	13.8	308.4
2022	1362.4	829.9	817.6	570.9	246.7	0.1	12.2	339.2
2023	1440.0	547.5	541.5	602.0	239.5	0.1	6.0	312.8

Source: own elaborations based on [40].

**Table 2:** Revenues and profits in the oil and fat industry in 2005–2021.

Parameter	Revenue (PLN Million)	Net Revenue (%)	Gross Profit (PLN Million)	Net Profit (PLN Million)
2005	2571	94.7	133	110
2006	3237	91.3	202	158
2007	3638	93.3	103	81
2008	5421	85.3	239	191
2009	7022	80.8	256	208
2010	4578.9	86.7	51.7	25.01
2011	4554.7	95.2	−10.8	−23.7
2012	6194	84	−17.3	−41.3
2013	6231.6	82	206.3	176.2
2014	5434	84.3	314.6	255.1
2015	4827	81.8	117.3	91.6
2016	4674	82.7	109.3	89.1
2017	4629	80.4	125.5	107.1
2018	4720	83.2	148.9	120.4
2019	4810	86.0	172.2	133.6
2020	6290	83.6	163.0	127.0
2021	8560	80.6	451.9	372.0
2022	13,289.8	75.4	519.7	451.8
2023	10,447.6	84	443.8	381.6

Source: own elaboration based on [38].

**Table 3:** Investments and investment rate in the fat industry.

Parameter	Investments (PLN Million)	Investment Rate (%)
2005	71.5	1.07
2006	76	0.94
2007	100	1.17
2008	117	1.17
2009	109	0.89
2010	75.8	0.75
2011	136.6	1.24
2012	292	1.44
2013	243	1.12
2014	204.7	1.03
2015	77	0.61
2016	93	0.86
2017	68	0.73
2018	97.2	0.71
2019	126.4	1.25
2020	160.0	1.45
2021	126.4	1.29
2022	201.7	1.85
2023	232.9	1.96

Source: own elaboration based on [24].

**Table 4:** Short-term and long-term debt in the fat industry.

Parameter	Short-Term Loans (PLN Million)	Long-Term Loans (PLN Million)
2005	380	26.04
2006	437	50.7
2007	296	103
2008	857	242
2009	585	225.9
2010	457.9	209.7
2011	499.6	121.7
2012	1218	481.4
2013	755	186
2014	742	169
2015	162	265
2016	92	271.7
2017	112.1	194
2018	180.2	253.2
2019	239.3	312.3
2020	295.4	214.8
2021	253.9	198.4
2022	2220.9	168.4
2023	1813	152.3

Source: own elaboration based on [24].

**Table 5:** The results of the multiple regression analysis describing the relationship between the dependent variable Y_1_ (gross profits in the fat industry) and independent variables.

Variable	Regression	Error	Student’s *t*-Distribution	*p*-Value
Absolute term	14.443	4.357	3.315	0.000
X_1_—domestic rapeseed production	0.058	5.124	3.615	0.003
X_3_—margarine production	−1.200			
X_5_—current liquidity	−3.200			
X_6_—price of exported rapeseed	−0.060			
X_7_—monthly consumption of rapeseed oil	4.11			
X_8_—monthly export of rapeseed oil	0.031			

Source: own elaboration.

**Table 6:** The results of forward stepwise regression describing the relationship between the dependent variable Y_1_ (gross profits in the fat industry) and independent variables.

Variable	*β* *	Standard Error	*β*	Standard Error	*t* (14)	*p*-Value
Absolute term	−	−	18.851	5.215	3.615	0.003
X_1_—domestic rapeseed production	0.058	0.032	0.0036	0.0019	1.7952	0.0959
X_3_—margarine production	−0.1217	0.0415	−0.039	0.0133	−2.930	0.0117
X_5_—current liquidity	−3.1995	1.0917	−3.1516	1.0753	−2.9307	0.012
X_6_—price of exported rapeseed	−0.057	0.027	−0.0097	0.0046	−2.1107	0.0547
X_7_—monthly consumption of rapeseed oil	4.1098	1.0781	4.0100	1.0519	3.8120	0.0022
X_8_—monthly export of rapeseed oil	0.031	0.0217	0.0000	0.0000	1.4284	0.1767

Key: *β*—regression coefficient, *β* *—standardized coefficients, *t*—Student’s *t*-distribution, *p*—significance threshold. Selected regression statistics: multiple R = 0.998, multiple R^2^ = 0.997, adjusted R^2^ = 0.996 F (6.13) = 939.81, *p* < 0.00, standard error of the estimate = 1.2718. Source: own elaboration.

**Table 7:** Partial and semi-partial correlations between the dependent variable Y_1_ (gross profits in the oil and fat industry) and independent variables in the regression model.

Variable	*β* *	Partial Correlation	Semi-Partial Correlation	Tolerance	R^2^	*t* (14)	*p*-Value
X_1_—domestic rapeseed production	0.0577	0.4457	0.0238	0.1709	0.8290	1.7952	0.0958
X_3_—margarine production	−0.1217	−0.63077	−0.0389	0.1025	0.897	−2.931	0.0116
X_5_—current liquidity	−3.1994	−0.6307	−0.0389	0.000	0.999	−2.930	0.0116
X_6_—price of exported rapeseed	−0.0570	−0.5052	−0.0280	0.2426	0.7573	−2.1107	0.0547
X_7_—monthly consumption of rapeseed oil	4.1098	0.7265	0.0507	0.000	0.9998	3.8120	0.0021
X_8_—monthly export of rapeseed oil	0.0311	0.3683	0.0190	0.3728	0.6271	1.4284	0.1767

Source: own elaboration.

## Data Availability

The original contributions presented in the study are included in the article, further inquiries can be directed to the corresponding author.

## Nomenclature

EU	European Union
EUR	Euros
GHG	Greenhouses Gasses
GDP	Gross Domestic Product
ICT	Information and Communication Technology
PLN	Polish Zloty
RED	Renewable Energy Directive
RES	Renewable Energy Sources
SDG	Sustainable Development Goals
